# Sedated beauty: The invisible knife in online narratives about cosmetic breast augmentation

**DOI:** 10.1177/13634593241270950

**Published:** 2024-09-16

**Authors:** Petra Roll Bennet

**Affiliations:** Stockholm University, Sweden

**Keywords:** ethnography, gender and health, narrative analysis

## Abstract

Breast augmentation is a prevalent cosmetic surgery procedure among women in Western societies, and the cosmetic surgery market has witnessed substantial growth. Today, websites and online forums are platforms that feature discussions about cosmetic procedures. A genre on surgery clinic websites is ‘patient stories’, but also lay-initiated internet forums facilitate discussions and shared experiences related to cosmetic surgery. This study aims to analyse lay-initiated online narratives about cosmetic breast augmentation. The shared narratives contain descriptions of how women who are about to undergo breast augmentation prepare for surgery, the medical procedures that take place on the day of surgery itself, and the experiences and feelings after waking up after anaesthesia. Employing a structural analysis of 30 of these stories, this research illuminates how the surgery stories adhere to a conventional storytelling format, and how key characters within the stories are ‘helpers and makers’, including relatives, nurses and surgeons. The focus in these narratives revolves around the woman herself, although her active involvement is primarily observed during the preparation phase, with a more passive role assumed during subsequent clinic routines. Despite instances of pain and discomfort in the narratives, the stories are enveloped in an aura of glamour and a spa-like atmosphere. It is discussed how this ‘fairy tale’ story, narrating a surgical metamorphosis, seems to align with the popularisation of the cosmetic surgery sector.

## Background

‘Finally, it’s my turn to have breast surgery! This is my story’. This opening statement frequently marks the beginning of what is commonly referred to as ‘surgery stories’ (*operationsberättelser* in Swedish) found on a Swedish online forum dedicated to discussions about breast augmentation through cosmetic surgery. Similar so-called ‘patient stories’ have also become a common feature on cosmetic surgery clinic websites, where women share their personal testimonials about their surgical experiences, but in a more commercial environment (see, e.g. Akademiklinken, 2023; The Private Clinic, 2023). In 1991, Morgan, in her classic text *Women and the Knife: Cosmetic Surgery and the Colonization of Women’s Bodies*, predicted that elective cosmetic surgery would become increasingly prevalent. Morgan’s (1991) text aimed to explore ‘the knives that “sculpt” our bodies to make us beautiful forever’ (p. 26) and discussed the ongoing normalisation of cosmetic surgery. Mercer (2009) asserts that media hype and professional incentives have contributed to public expectations, reaching a critical point, and culminating in a ‘perfect storm’ within the cosmetic surgical market (p. 215). A decade later, this normalisation and ‘storm’ persists, with the cosmetic industry now flourishing. Official statistics on cosmetic surgery in Sweden are not yet available; however, according to the Breast Implant Register Annual Report (BRIMP, 2021), 11,000 cosmetic breast augmentation procedures were registered in 2020 and 2021. According to the International Society of Aesthetic Plastic Surgery (ISAPS, 2022) Global Survey 2022, breast augmentation remains the most common surgical procedure for women, with 22 million procedures and a significant increase of 29% compared to 2021. Cosmetic surgery is not offered as part of Swedish public healthcare, and breast augmentations are almost exclusively performed at private clinics. The industry has received attention for being untrustworthy and a new law has stated that aesthetic surgical procedures must be performed by a licenced medical doctor specialist competent in a speciality appropriate for the procedure. There is also an age limit of 18 years for treatments (Law, 2021:363).

According to Fraser (2003), cosmetic surgery discourse relies heavily on repertoires of agency and how cosmetic surgery is seen as a ‘positive career move, and the participant (is) an active, motivated and intelligent person’ (p. 35). Pitts-Taylor (2009) discusses how the cosmetic industry employs arguments that align with the current neoliberal agenda, that emphasises personal choice and self-empowerment, promoting notions such as ‘you are worth it’, and ‘you have the autonomy to pursue self-improvement’. According to Dolezal (2010), cosmetic surgery procedures are framed as ‘an empowering decision, not solely about appearance, but self-care’ (p. 371). Moreover, the industry emphasises the idea of achieving harmony between one’s inner and outer self (as seen, e.g. on Swedish clinic websites such as Akademiklinken.se). Undergoing cosmetic surgery has been described as an experience that represents empowerment and self-improvement (Davis, 1995), but has also been reported as a questioned experience, linked to moral, political or psychological vulnerabilities (Saxena, 2013; Pitts-Taylor, 2009), and connected with personal fears of being seen as vain and shallow (Roll Bennet, 2022). Bonell et al. (2021) identify a tension between the encouragement to undergo cosmetic surgery and the condemnation of it, which they refer to as the ‘cosmetic surgery paradox’. They claim that the ‘medicalization of physical appearance serves as a moderator in the relationship between unattainable female beauty standards and the popularization of cosmetic surgery; that is, the relationship is stronger when appearance is medicalized’ (Bonell et al., 2021: 236). The ‘paradox’ of the cosmetic surgery might be managed by participating in an online community forum dedicated to cosmetic surgery. Such a forum can be a ‘safe’ environment for women who are considering breast augmentation. Participation allows for the sharing of questions, doubts, hopes and support from others in similar positions or, even better, more experienced members. Findings from studies of health-related online forums suggest that these can become ‘safe’ spaces where individuals share experiences with others who have had similar experiences (Hanna and Gough, 2016) and be a place for challenging established health institutions by questioning the dominance of the medical and health sector (MacLellan, 2022; Satija, 2022). Dym et al. (2019) showed how online communities provided support for LGBTQ+ people coming out and how narratives led to identity recovery work, concluding that stories told in forums may have an agentic function. Massa and Simeoni (2017) showed that forum pregnancy stories, ‘represent an expression of agency and creativity’ (Massa and Simeoni, 2017: 140) and argued that the opportunity to create a space for sharing personal issues with others in the same situation lead to the creation of communities for support, and that ‘people often tell stories to work out their own changing identities, giving voice to an experience inadequately described through the dominant discourse and to guide others who will follow them’ (MacLellan, 2022: 183). This present study aims to explore the sharing of experiences within women’s first-hand online stories about breast augmentation through cosmetic surgery by exploring the content, the narrative structure and by describing the key figures in the narratives. Additionally, the form and content of these online stories will be discussed in relation to the popularisation of the cosmetic industry.

### Cosmetic surgery research

Understanding why so many women are attracted to cosmetic surgery is a key question for feminists (Heyes and Jones, 2016) and feminist cosmetic surgery scholars began with a significant critique of the patriarchal medicalisation and normative disciplining of women’s bodies (Bartky, 1997; Bordo, 1989; Morgan, 1991). This ‘early’ feminist writing posited that the cosmetic industry operates as a mechanism of control, perpetuating the subjugation and oppression of women. However, some scholars have countered this viewpoint by highlighting the importance of acknowledging women’s agency in the context of cosmetic surgery (Davis, 1995). Davis argues that an exclusive focus on the controlling aspects of the cosmetic industry may position women as passive recipients of societal norms and as ‘cultural dopes’, undermining their capacity for autonomous decision-making. Also, Pitts-Taylor (2009) challenged conventional notions of agency when researching cosmetic surgery. She contended that understanding these practices requires an examination that goes beyond purely internal or external perspectives, emphasising instead the intersubjective nature of the subject’s agency (Pitts-Taylor, 2009: 122).

Research commenting on the beauty and cosmetic industry has explored the development of the female body as an object depending on various body practices and activities aimed at attaining the ‘normate’ standard imposed by the fitness, beauty, fashion and diet industries (Dolezal, 2010). Also, Hurst (2015) has defined cosmetic surgery as a seductive fantasy that promises that bodies are limitlessly transformable. According to Dolezal (2010), these beauty industries tend to emphasise external solutions and products to achieve the desired body aesthetics, perpetuating the notion that almost everything can be bought, often at an affordable cost. Dolezal highlighted a paradigm shift wherein the pursuit of the ideal body had become intertwined with medical practices, transforming beauty into a medical concern. An example of this intertwinement is Taylor’s (2012) study which investigated the experiences of young women who underwent breast augmentation surgery. Her research revealed that these women exhibited varying perspectives on the procedure, oscillating between viewing it as a simple beauty enhancement and a medical intervention. Cosmetic surgery sits at the crossroads of medicine and consumer society dynamics, and is one of the most commercially influenced medical fields (Voinea, 2018). This central ambiguity of the cosmetic surgery procedure’s position between the medical and the cosmetic is evident in the field of so-called ‘medical tourism’. Research on cosmetic surgery tourism (Holliday et al., 2019) shows how both those seeking and paying to change their bodies, the ‘medical travellers’, as well as surgeons, nurses and others working at the clinics, can be seen to exist in a borderland between healthcare and a spa or beauty salon. Ackerman’s (2010) ethnographic study demonstrated the significance of the location, how the entire stay in a ‘healing landscape’ and how the interactions with those working there and for the women undergoing surgery contributed to a sense of exclusivity and selectiveness, and how the entire surgical experience was perceived as an adventure and a break from ordinary life. Important research of Gimlin (2012, 2013) has explored rhetorical resources used by women to explain their decision to have cosmetic surgery, and Gimlin show how women align with notions of legitimate medical treatment.

### Transformation narratives

This present study focuses on women’s first-hand online narratives. Narratives, or storytelling, are a fundamental part of human communication, because human beings wish to create connections between events and meanings of everyday life mediated through narrative forms (Ricoeur, 1984). Narratives are fictional statements that, to varying degrees, are about real lived lives (Denzin, 1989). Research into narratives can involve asking questions such as how and why the narratives are structured the way they are, to whom the story is told and for what purpose, and what the story accomplishes (Riessman, 2008). Research on narratives of body change and transformation has, among other things, examined illness narratives (Frank, 2013; Hydén, 1997), and studies on transformation through surgery have, for instance, revealed experiences of weight-reducing surgery (Throsby, 2008). Thorsby followed, but not systematically, an online forum discussing experiences of weight-reducing surgery and found that the features of the forum were very positive and encouraging, a ‘happy place’, where shared experiences of ‘failures’ were rare. The above illness narratives share similarities with narratives about cosmetic surgery, but are more clearly rooted in a medical context, and differ regarding the necessity of the treatment. This question about ‘necessity’ and reasons for why the surgery is performed becomes even more evident when compared with narratives about surgery due to breast cancer, such as reconstruction after a mastectomy (Sandell, 2008). The surgical practice is similar to the breast augmentation played out at the cosmetic surgery clinics, but in a completely different environment and for different reasons.

Research using a narrative perspective on breast cancer surgery is quite common (see, e.g. Rosenblatt, 2006; Thomas-MacLean, 2004) but a narrative approach to cosmetic surgery experiences is not very common. Most studies employing a narrative approach tend to focus on television shows like *Extreme Makeover*. For example, Heyes (2007), in her analysis of television makeover shows, found that the makeover shows rests on a narrative based on fairy tales and that ‘the fairy tale is a useful heuristic because the generic, supposedly universal quality of the characters and plot draws attention away from the political messiness of real-life transformation’ (Heyes, 2007: 27). Lee (2009), in her analysis of makeover shows, analyses how the narrated ‘tales’ include various characters whose function is to assist the women in some way, with surgeons being identified as the most important figure. Lee found that the surgeon’s role transcends that of mere operator; he (it is often a male) emerges as a creator of beauty and an agent of transformation who modifies women’s bodies; in cosmetic surgery (in a somewhat ‘Cinderella’ narrative) the surgeon’s hands are seen to possess a kind of ‘magic’ ability that accord them transformative power. Pitts-Taylor (2007) analysed *Extreme Makeover*, describing how the surgeon had awakened ‘the sleeping beauty within her’ (p. 51).

## Research material

The narratives analysed in this study come from a Swedish online forum that focuses on discussions related to all cosmetic surgery procedures. Notably, the forum was established by individuals who had personally experienced negative encounters with cosmetic surgery, and it operates independently from any specific surgical clinic, but the primary source of revenue for the forum is derived from advertisements for various surgery clinics. The forum functions on an open-access basis, allowing one to read posts without any restrictions, but active participation and contribution to discussions require (free) membership of various membership levels, with some granting access to viewing and sharing pictures, depending on whether one is active and posting new threads. The third level, which the surgery stories are part of, is for users who have been members for at least 15 days and share information about personal details such as age, height, weight, number of children, the upcoming surgical treatment, the selected clinic and the surgeon. Most women indicate their age, with a median age of 25, encompassing a range from slightly under 20 to just over 50. Common topics on the forum include seeking advice before surgery, sharing experiences about different surgeons and clinics, and inquiring about post-operative healing processes. In terms of the tone and atmosphere, the forum is characterised by a high level of politeness and encouragement among its participants. The topic labelled ‘Surgery stories’ has been a focus of the forum since its inception in 2012 and over a thousand stories have been published since then. The most common procedure in the surgery stories is breast augmentation, followed by liposuction and rhinoplasty. While the quantity of surgery-related stories has declined since its peak in 2015, new stories continue to be posted weekly. The stories between 2015 and 2020 are mostly written in a cheerful tone, but the stories from 2020 to the present are more precise and correct in comparison, with a more distant tone. This change might be attributed to the fact that forum followers are getting older, and younger women in their 20s have followed more visual forums or influencer sites where videos are available.

As a researcher, I have been following and reading posts on the forum since 2019, familiarising myself with the form, structure and content of the narratives. To get an illustrative and manageable sample of stories to analyse, I did an in-depth reading of every second surgery story about breast augmentation between 2015 and 2018. This process resulted in 114 stories, each approximately 650 words, with some shorter or longer exceptions. In this first reading, I noted how the stories were written in a particular way, with similar content and structure (see the typical story below). I then randomly chose 30 stories from the original sample for a structural analysis (Labov, 1972). Subsequently, I incorporated 20 more stories from between 2020 and 2022 to ensure a more updated and thus relevant sample of narratives.

### Ethics

The research ethics around using online data are still being worked out, with some arguments claiming that no consent is required since the data are in the public domain (Drioli-Phillips et al., 2021). My ethical considerations follow precedents for use of this kind of material (Bennett and Gough, 2012); and following Hanna and Gough (2016), the usernames and other information that could identify individuals have been anonymised, no story is quoted in its full length, and the quotes are translated from Swedish into English, making them unsearchable. It is important to note that because the study engages with a large number of texts and not the uniqueness of the individual story, it is the stories and not the individual narrators that are the focus of the analysis. The research project has been approved by the ethics support function at the Office for Research, Engagement and Innovation Services, Stockholm University.

### Analysis

Following MacLellan (2022), this study employs a narrative analysis rooted in Labov’s (1972) structural narrative analysis, and aims to examine the narrative in terms of how stories are organised by the writer, and why (Riessman, 2008). Adhering to Labov’s methodology involves the preservation of substantial narrative segments. Labov (1972) assumed that narratives are stories about a specific past event, having common elements that together make a ‘fully formed’ narrative that includes six common elements: an abstract (summary of the substance of the narrative); orientation (time, place, situation, participants); complicating action (sequence of events); evaluation (significance and meaning of the action, attitude of the narrator); resolution (what finally happened); and coda (returns the perspective to the present).

This study used these six narrative elements to analyse the structure of the stories about the day of the surgery to provide a view of the plot, the strategic interplay of narrative components, and the narrative action (Riessman, 2008). According to Labov (1972), a detailed analysis can help reveal the function and meaning of a narrative, and narrated events become meaningful partly due to their placement in the narrative (Riessman, 2008). Based on what emerged in this structural narrative analysis, I chose to proceed with a more in-depth analysis of the various characters described in the story. Using the concept of ‘roles’ within the narrative (Herman, 2007), I decided to look closer into how the presented characters were described and how their actions and functions are presented in the story.

## Findings

First, I introduce a typical story that recurred across the sample narratives, covering the prevalent and recurrent elements commonly found within its content. To preserve ethical considerations, the story is composed and derived from a wide-ranging sample of stories to ensure author anonymity. In the following analysis, additional quotations are added for more clarity and to deepen the content. These selected elements are intact and translated from Swedish. My typical story is presented in accordance with Labov’s (1972) structural approach, with sections which I determined, following and using Labov’s terminology.

### A typical story

*Abstract* (summary of the substance of the narrative): Finally, it is my turn to write my story about the surgery! It has been so exciting to read yours!

*Orientation* (time, place, situation, participants): I will take it from the beginning.

*Complicating action* (events of the narrative, the occurrences that move it ahead): The night before the operation, I changed all the linen, took a long shower with Descutan [antibacterial soap], and went to bed early because it was a long drive the next day. Next morning, I woke up early after an ok sleep, took the last Descutan shower, put on soft, clean clothes and braided my hair. Then me and my partner started the journey towards Stockholm. Considering the snow chaos, we went at half past eight, although I wasn’t supposed to be there until 11.

*Evaluation* (significance and meaning of the action, attitude of the narrator): I was a bit nervous when I went to the reception.

*Complicating action*: I was welcomed by a very nice girl and was assigned a room of my own; Room 8 it was. It was a very nice room and a cosy bed. I really liked the view over the water! I put on a soft dressing gown and slippers. I had to wait a while before a nurse came in and gave me some pills to swallow with very little water. The pills I took were for nausea and painkillers because of pain after the surgery.

*Evaluation*: The nurse was so nice and cheerful and made me feel comfortable.

*Complicating action*: After a while, [name of surgeon] came in and drew on me; it was very quick. He was a bit like an artist, with pretty fingers and a small measuring stick, it took maybe 2 minutes. We talked about the size I had chosen and the shape; it was [name of the brand], round shape 450 cc behind the muscle.

*Evaluation*: When he left, I understood that it was my turn soon. But I still wasn’t very nervous. So, a nurse walked in and said it was time to head to the operating room. And that’s when it hit me – I realised what was actually about to go down!

*Complicating action*: The nurse led me to the operating room which had a completely different vibe compared to the cosy atmosphere before! It was a bit on the chilly side, but there was music playing in the background. I hopped onto the hospital bed, which was surprisingly comfortable and cushy, and they draped me with this warm, heavy blanket. My arms were strapped to my sides and the famous [name of anaesthetic doctor] was nice and joking and put a PVC in one of my arms and told me to think about something nice, and before I knew it, I fell asleep.

*Resolution* (the outcome of the plot): I remember it was half past two when I was taken into surgery; when I woke up, it was half past three, crazy fast! I remember that I had no pain at all and felt great, but the pain only increased from there, almost unbearable; the skin was stretched so much! I felt a bit dizzy and nauseous, especially when I heard other girls vomiting and crying nearby, poor them! But then I looked down at the breasts and just lay there with the biggest smile! I saw a cleft! I finally have boobs!

*Complicating action*: Soon after that, I was helped to my room, and then I called my partner and my mum and told them that I was fine. Then the finest tray came in with a good cup of coffee, a delicious sandwich, vanilla yoghurt with granola, blueberries and mango, and a glass of juice – YUMMY!!!!! Then it was time for a visit to the toilet, dizzy and a little shaky; but I was accompanied to and from by a sweet nurse. Then I dozed until 5:00 pm. My partner came and picked me up, and we drove home. I had painkillers every 6 hours and tramadol for the night, so I slept quite well.

*Coda* (returns to the perspective of the present): I can really recommend the clinic, and [name of the surgeon] is fantastic; my breasts are perfect (I think, haven’t seen the final result yet, but they look fantastic!) I am so happy I finally did it! That was my little story – HUGS to you all!!!!

### A structural analysis

The story has an introduction that addresses actions of preparation, going to the clinic, short evaluations about feelings of being nervous or not, complicating actions just before and after the operation, and going home. The surgery stories follow the classical form of storytelling (Riessman, 2008), where the progression is structured chronologically, tracing events and occurrences through time. Looking more closely at the elements, the introductory *abstract* is commonly very positive, with exclamation marks, and typically adding that it is her turn to write the story. The presented *orientation* is the most common in the stories, proclaiming the first scene which is the first *complicating action*. It starts either the day before or the morning of surgery. The complicating actions involve what can be viewed as rituals characterised by preparation elements. The rituals often include changing the sheets, sometimes those of the whole family if there is one, showering with a special antibacterial soap that is always mentioned, brushing the hair carefully, making a ponytail or braid, using body lotion and putting on clean, soft clothes, as illustrated by the narration below:

The night before the surgery, I took a shower using [name of anti-bacterial soap] right before hitting the hay. I made sure everything was set – fresh sheets, clean clothes – you name it. I gave the whole place a good cleaning and stocked up the fridge. I dressed comfortably in stretchy pants, a brand-new zip-up hoodie, and a sports bra that hooks in the front. Packed in my bag were pyjamas with buttons, cosy slippers, face cream, a toothbrush, and some hand and lip moisturise. I braided my hair, all ready to go.

The preparations at home and the journey to the clinic are often described with a cinematic shimmer – magical and full of rituals concerning the preparation of the body. The preparation is characterised by cleansing and purifying rituals; almost as if one needs to cleanse oneself of impurities in preparation for a transformation. In addition, it is about being on time and bringing what you might need. *Evaluations* are relatively scarce, and when present, in these first phases, it typically focuses on whether the women are anxious. The *complicating actions* that follow arrival at the clinic start with meeting the first character at the clinic, who is always a nice, friendly nurse at reception

Once inside the clinic, I was well taken care of, I was referred to the care department where I was met by a super nice nurse who led me to my own room. with my cosy bed and TV, of course. I was shown my dressing gown, socks and slippers and asked to take a shower immediately (with special soap) as I was the first one out for the day. What a fancy bathroom!

This phase is described as a bit ‘luxurious’ warm, and cosy, and involves changing into clinic attire, settling into a comfortable bed, often commenting on the nice surroundings. This is also a phase of waiting for a pivotal moment: the entrance of the surgeon, who is described as exuding both confidence and professionalism, and being in hurry. The surgeon’s task in this phase is to draw markings on the chest, and discuss and decide the size of the implants:

We proceeded to [name of the surgeon’s] room, where he photographed my breasts, conducted measurements, and made markings on my chest. Two days prior, I had communicated my desire to switch to a high-profile implant. However, his uncertainty about availability prompted a suggestion to alter the plan from 280cc to 320cc. I agreed, albeit with some subsequent reservations, as mid-profile implants are known for their broader dimensions. My concern lies in their potential width, yet I am happy with my decision to opt for the larger size.

This procedural sequence is commonly told in a formal narrative style, characterised by an objective description of the unfolding events. The shape and size of the chosen implants are consistently communicated, and is sometimes a discussion between the surgeon and the women about achieving the optimal outcome. This meeting is typically narrated with a professional tone, without describing any sensory or emotional discomfort tied to the act of being marked and sometimes photographed for before- and after-pictures.

The story has now been built up from the preparation phase, the waiting, and the meeting with the surgeon, and now follows the second *evaluation* that is placed strategically to signal feelings of nervousness or not before the next *complicating action*, which is the most significant: the surgery phase. The soothing atmosphere quickly shifts to a clinical, cold and rigid environment during the swift pre-surgery preparations. The change in the atmosphere is signalled by descriptions of the operating theatre as cold; the arms are strapped to the sides, and everything happens quickly:

The nurse stuck a needle in the bend of my arm and hooked me up to this machine. She pointed out that my low blood pressure, which I had mentioned on the health form, was indeed true – just like it always is. The anaesthetist and the nurse teamed up and did all the medication stuff through that arm needle. They even slapped an oxygen mask on me (which, FYI, smelled pretty rubbery). And before I knew it, I was out like a light.

This colder and clinical moment contrasts with the preceding events. However, the bed, frequently ‘hopped’ onto, offers a pleasant and cosy contrast. While the surgeon is rarely mentioned in this context, a cheerful anaesthetist often assumes the main role and helps her to sleep. After the surgery, which can be said to be the story’s plot, follows the *resolution*: The experience is often commented on as if no time has elapsed, with frequent evaluations about pain or its absence. Descriptions of sensations of nausea, coldness and drifting into slumber are common, as are comparisons of their discomfort or well-being to the sounds of other women crying out in pain.

The narrative culminates as the objective is achieved – the breasts are now larger. The next described complicating action is the post-surgery phase, involving actions such as reaching out to friends and family, receiving a thoughtful tray of nice food, gathering oneself and being picked up to head home. The *coda*, with very few exceptions, narrates an experience that was excellent. There are no regrets, and the clinic’s staff is praised. The tone is positive and happy, with a sense of accomplishment – everything went smoothly; finally, it is done, and it is perfect.

The surgical narrative might be viewed as a subset of a larger genre, encompassing both its structure and content drawn from elements of fairy tales such as Cinderella or Sleeping Beauty. The surgery narratives also borrow classical elements and dramaturgy from other stories, such as giving birth (MacLellan, 2022; Sanders, 2019) and pregnancy stories (Massa and Simeoni, 2017). Similar to the pregnancy tales, cosmetic surgery narratives also depict associated rituals. These include the preparation phase and the sense of relief and joy that follows enduring the painful yet essential procedure, resulting in a remarkable transformation and a deep sense of happiness. A typical element is the presentation of a carefully curated food tray after the painful phase, providing the woman with nourishment and a form of reward. Linguistically, the stories are a mixture of words denoting soft and warm features and a somewhat medical language that helps the content and form work together to accomplish the stories’ aims. These narrative models often involve the concept of sleep, the presence of a magic prince (the surgeon) and the theme of awakening or rebirth. The transformative journey in these stories allows the protagonist to embrace a new identity, aligning with her true self and fulfilling her destined purpose.

### Characters’ roles and actions

Turning to the analysis of roles, I will describe the different characters in order of their appearance in the story. The first character is the woman herself, who is the one preparing, waiting and being helped and transformed. The main character, the woman herself, takes on different personas as the narrative unfolds. In the preparation phase, she demonstrates both effectiveness and a touch of nerves, while on the brink of surgery, she is filled with a blend of nervousness and anticipation. As the story progresses, the audience is given descriptions of her physical sensations, including pain and dealing with nausea, and now needing support to get through the process. After overcoming the aftermath of the surgery, she gathers herself to head home. The narrative unveils a woman seizing control, as her choice to undergo surgery displays a firm determination.

The stories then include several people who can be defined as ‘helpers’ and ‘makers’. The helpers can be mothers accompanying their daughter, giving her a ride to the clinic and sometimes following until just before surgery. In some cases, the partner, ‘boyfriend’, does the same. These helpers are described as necessary and company, being there before and after entering the clinic:

The evening before the operation, my partner and I went home to my mother and her husband. We planned to sleep over there just so mom could drive me to the clinic the next morning. The alarm rang at 05:15 the next morning when my partner was leaving for work. So we said goodbye to each other, and then he left, but, of course, I couldn’t sleep after that. The day had finally come, after 3 months of waiting! Time passed quickly, and suddenly, me and mum were in the car on our way! When I got there, I registered, and I was helped right away and shown to my room by a nurse. Mum came with me to the room and wanted to join in, so she also got to hear all the information from the nurse. After that, mom drove home.

Inspired by Lee’s (2009), the ‘assisting characters’, the makers, represent the characters at the clinic: nurses, surgeons and anaesthesia staff. The first to enter the scene is the nurse at reception, who is consistently portrayed as welcoming and kind. Nurses are described as always available and supportive but are rarely, almost never, mentioned by name. The relationship with the nurses is portrayed as close: she is touching the woman’s body, helping and assisting, especially after surgery, when the woman might need help with nausea, pain and the toilet:

I had to wait another hour or so before the nurse came in with painkillers. Together, we went to the operating room. There was some music playing, cheerful nurses dressing me, talking about how everything was going to work, joking that I could be having crazy dreams and that I shouldn’t worry about the pressure many women feel when they wake up. Again, they were so cute that I almost had tears in my eyes, ha-ha. Woke up around 3pm and was so shocked because I didn’t understand anything, had two nurses calm me down a bit, started to break into a cold sweat (which is normal), so I focused on breathing, and a nurse cooled my face for about 10 min. Had to go and pee and was helped by the nurse and felt dizzy and tired. I wanted to sleep, but she said she would like me to eat something, so I had to eat a good sandwich.

Because the nurses are consistently depicted as nice and helpful, it may position them as both helpers and makers, working to keep the woman in a good mood before the surgery and assisting her afterwards to make sure she can leave the clinic. The primary maker in the story appears to be the surgeon, whose name is always emphasised, and he (it is often a male) plays a significant role. The surgeon has a room of his own where he assists in selecting the implant size and draws markings on the woman’s chest before the procedure. The surgeon sometimes, not seldom, recommends larger implants:

I waited a while in my room before [name of surgeon] picked me up to draw on me and check that we agreed. I started talking about wanting a mini or demi and pretty low ml but [name of surgeon] laughed a little and said straight and clearly (but nicely), ‘If you want the breasts you sent pictures of, you should go up in size. For your body type, Demi 360–400 will be just right based on the small firm breasts you are looking for. You shouldn’t think about ml here and there, but you have to consider how the body looks. Mini also works on you, of course, but then you won’t get the same as the pictures’. He was so convincing, and it made me feel safe – he is the expert. ‘My God’, I thought ‘am I going to get such large implants?!’ But I felt really safe.

This passage holds multiple points of interest. It carries a somewhat dramaturgical essence, with the surgeon displaying notable engagement in determining the optimal breast size for the woman. Such reported conversations like this one are a recurring feature in the narratives, underscoring the communication between the surgeon and the women. The surgeon is presented as a proficient authority on breast size and the contours of the women’s bodies and a reliable and trustworthy expert. Interestingly, the envisioned transformations often result in larger breast implants than initially anticipated, and after this stage, the surgeon departs, leaving no further trace.

## Discussion

This study aimed to explore the content and narrative structures within women’s first-hand online stories about breast augmentation through cosmetic surgery. The findings show how the narratives are written in a ‘classical’ narrative form and structure, making the story into an engaging and convincing script that may have a strong impact on both readers and authors, partly because the structure is so similar to that of typical storytelling (Riessman, 2008). The narrative elements and their content support the established genre in the forum, a consistency that also may function as a reassurance of a predictable experience. This could also be part of the need to demonstrate an ability to tell the story the ‘right way’, to prove that one is part of the community, that one is worthy of full informal membership (Massa and Simeoni, 2017).

Regarding the narrative content, the different phases are embedded in the presentation of a spa-like environment, except for the short time before and after surgery, where the discomfort is quickly alleviated by rituals such as a fantastic tray of food and drink, similar to those offered to women after childbirth (Sanders, 2019). The mix of cosy, soft and sometimes glamorous elements with the harsher medical surroundings may serve to legitimise the experience (MacLellan, 2022) and is represented by the spa-like clinic, nurses, surgeons and the operation procedure, all together creating an air of assurance that can also function as arguments for the necessity of the experience.

Comparing cosmetic surgery stories with other transformative stories such as weight-reduction surgery (Throsby, 2008) or breast cancer surgery (Rosenblatt, 2006), the cosmetic transformation might be considered a more questionable surgical procedure (Bonell et al., 2021; Saxena, 2013). Riessman (2008) suggests that narratives often carry a moral aspect, and because narrative is used as a means to communicate the cosmetic surgery experience, a moral lesson could be expected in the online stories. However, within the specific narrative about breast augmentation, the moral lesson is not very clear. Instead, the story emphasises triumph and success, portraying the central protagonist in a safe and cosy environment, helped to stay calm during moments of nervousness and physical pain, and subsequently reaping the rewards. These mixed elements form a narrative reminiscent of those characterising the pregnancy experience (Massa and Simeoni, 2017), a story that underscores the adventurous essence of the birth process, as well as the rituals associated with the experience, punctuated by challenges and moments of joy. The cosmetic surgery stories contain few examples of pain and discomfort, and when these are described as severe, they soon ebb away. This finding can be further elucidated by Pitts-Taylor’s (2009) discussion of pain and risk in cosmetic surgery, where she argues that pain is framed as something to overcome because it is worth it, given that cosmetic surgery is presented as a personal accomplishment. The moral aspect of the cosmetic surgery stories leans more towards a ‘success narrative’, where the desired transformation is ultimately achieved.

In the discussion of agency in narratives within online forums, the notion of agency adopts a somewhat different character in the cosmetic surgery stories. This is partly because the narrator oscillates between being a patient and being a customer, each with different types of agency. Unlike the findings in studies by MacLellan (2022), Satija (2022) and Dym et al. (2019), the surgery narratives do not exhibit the typical form of agency that is more directed *against* a kind of oppressive issue; rather, they align more closely with the common procedures in the cosmetic industry. The stories are adaptive, aligning with the beauty industry’s standards, and often lack room for nonconforming viewpoints. Aligning *with*, rather than against, the described context raises questions about the potential influence of these forums and stories on other women’s decisions to undergo breast augmentation surgery.

The present characters, the ‘helpers and makers’, play different roles and have different functions. At the centre of the story is the woman herself, but except for the preparation phase, she describes herself as quite passive in her role – waiting, and following the routines of the clinic. The surgeon is at the centre of the story, with his ability to sculpt, and his elevated position. Voinea (2018) claims that this expertise of male surgeons, incorporating both medical expertise and aesthetic sensibilities, leads women to entrust their bodies to surgeons, to be reshaped according to his recommendations. This could be because the surgeon ‘assume[s] the role of the male observer’ (Lee, 2009: 517), representing an objective male gazer (Mulvey, 1975). The role of the nurse in cosmetic surgery is not well examined; studies tend to focus more on the role of the surgeon, who is described as the primary maker (Blum, 2003; Jones, 2016). At the beginning of my analysis, I also focused on the surgeon’s role, regarding him as the critical, apex maker. However, during the analysis, I was struck by the narration of the nurses’ role and actions. They are both helpers and makers, serving as nameless ‘enablers’ whose function is to stay close to the woman’s body and pay attention to her mood. The fact that the nurses (women) are not mentioned by name could be indicative of a patriarchal hierarchy that may still persist. This might reflect how nurses, in the views of the storytellers and among researchers, are not perceived as being as important as surgeons. Nurses’ roles and experience, described in Ackerman’s (2010) study of medical tourism in Costa Rica, show how the nurses express a sense of relief at having left the conditions at public hospitals to the practices of private medicine, where there is a more relaxed, retreat-like atmosphere and fewer medical procedures are performed. Ackerman (2010) found nurses handling of patients’ emotions ‘much like flight attendants in the early years of commercial airlines’, who wore more ‘female’ clothing than they do in public healthcare, which ‘enacts a gendered model of nursing that is now perceived as outdated’ (p. 415). I find that the role of the nurses as presented by the narrators need more research attention – they act as female helpers, who guide the woman, take care of her cut body, and do emotional work to cheer her up, which are all vital contributions that enable a somewhat ‘unnecessary’ and sometimes questioned procedure.

Finally, I will discuss how the form and content of these narratives might encourage and align with cosmetic surgery industry. The heading, ‘*Sedated beauty – the invisible knife . . .*’ makes a dual reference – alluding to the fairy tale of Sleeping Beauty and the subtly mentioned knife in the surgical narratives. Morgan (1991) delves into the symbolic significance of knives in women’s lives, contending that the knives in cosmetic surgery narratives pledge the sculpting of bodies. The heroine, or protagonist, is sedated as these enchanted knives work their transformative magic. It is vital to recognise that what happens when the protagonist slumbers are inherently brutal and laden with risks for complications. Hopner and Chamberlain (2020) rightly summarise this reality as follows: ‘The material reality of this surgery is that women’s breasts are sliced open, implanted with a synthetic foreign object, and stitched closed’ (p. 3). This statement illustrates the physical reality behind the surgical procedure, highlighting the invasive nature of these interventions. The findings also illustrate how beauty continues to be a medical concern, and that the ‘feminist theorist must maintain vigilance with regard to biomedical discourse’s hold on women’s bodies’ (Dolezal, 2010: 372). The surgical stories can therefore play a part in sustaining the industry, crafted to support the cosmetic industry while also offering women a vital sense of community and belonging. The surgical metamorphosis, combined with the medical context, may function as a transformative power that fuel the growth and development of the cosmetic surgery sector.
